# Supplementary material for: Enhanced Antibacterial Activity of Substituted Derivatives of NCR169C Peptide
Source: Int J Mol Sci. 2023 Jan 31;24(3):2694. doi: 10.3390/ijms24032694 (PMC9917201; doi:10.3390/ijms24032694)

Supplementary Figure S1

Structural models of peptides (listed in Table 1.) predicted by AlphaFold (ChimeraX version 1.5)

A. NCR169C$_{17–38}$

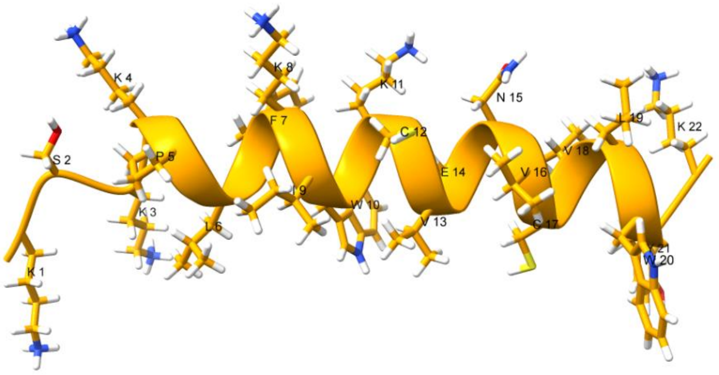

B. NCR169C$_{17–38}$ C$_{12,17}$/S

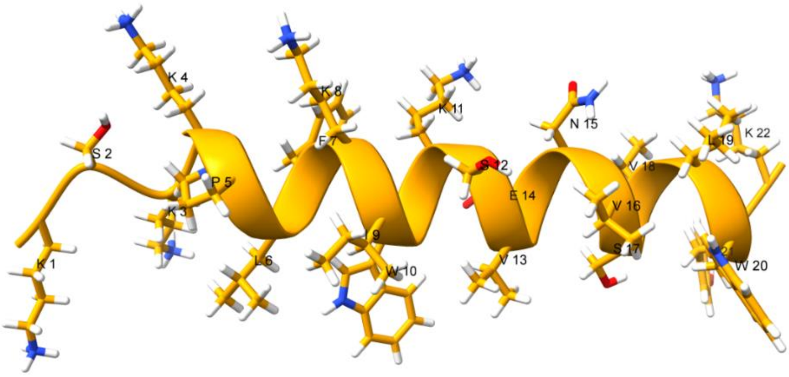

C. NCR169C$_{17–38}$ W$_{10,20}$/A

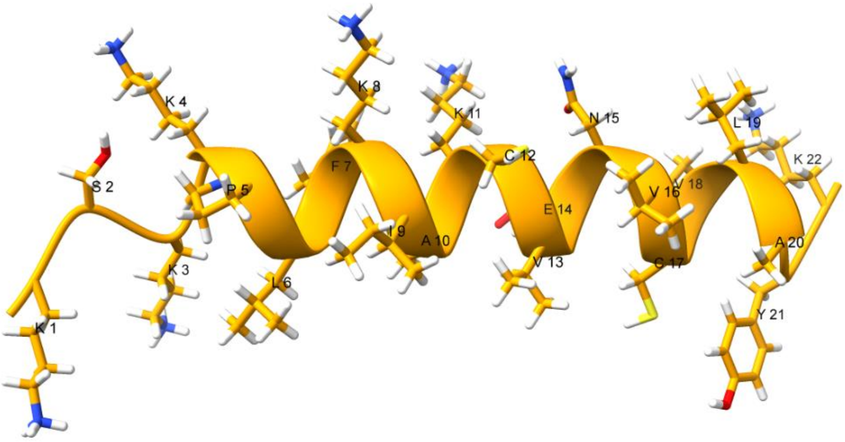

D. NCR169$C_{17–38}$ $W_{10,20}$/A, $C_{12,17}$/S

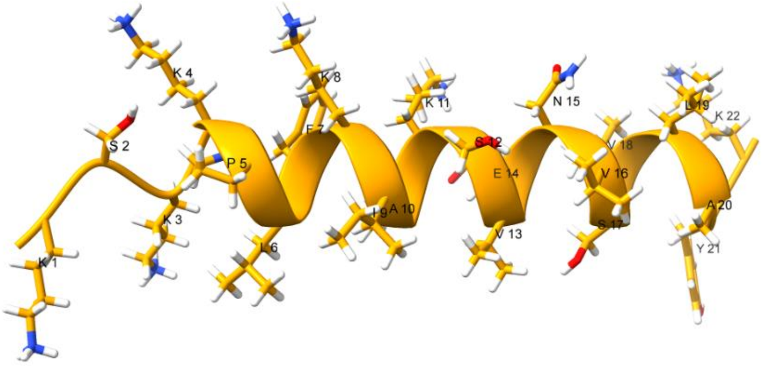

E. NCR169$C_{17–38}$ $W_{10,20}C_{12,17}$/A

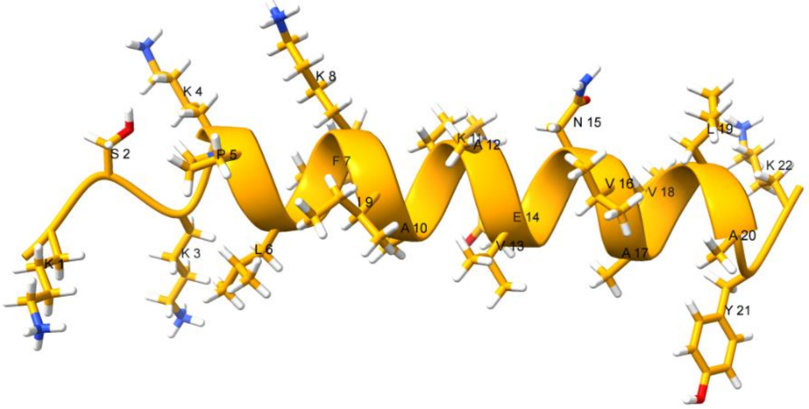

F. Full length NCR169

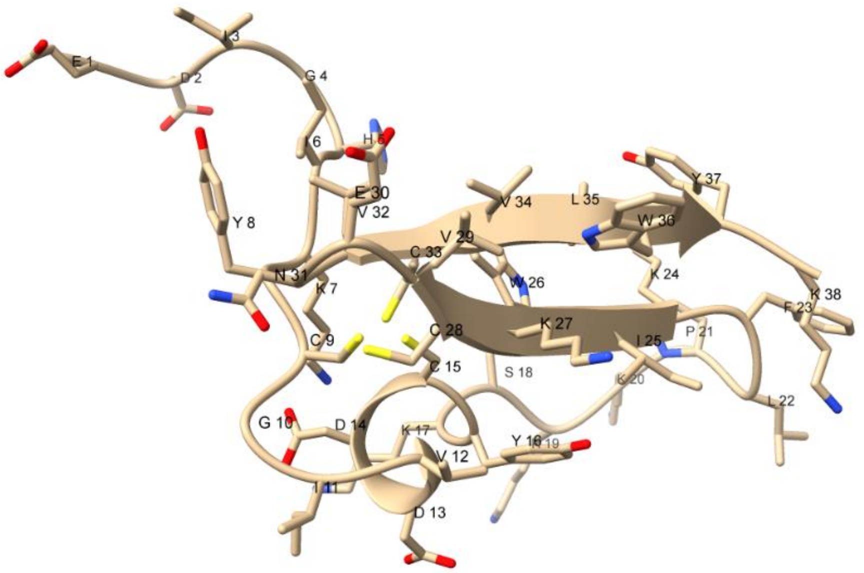

Supplement: Supplementary file 1 [file ijms-24-02694-s001.zip › ijms-2195045-supplementary.pdf]
